# Effectiveness of community—based Baduanjin combined with resistance training on cardiometabolic risk factors in older adults with type 2 diabetes: a 16-week randomized controlled trial

**DOI:** 10.3389/fendo.2026.1930328

**Published:** 2026-08-27

**Authors:** Fengfeng Liu, Xiaoxiang Zhu, Haokai Leng, Changzhou Chen

**Affiliations:** 1 College of Physical Education, Shanghai University of Sport, Shanghai, China; 2 Preventive Medicine, Juquan Xincheng Community Health Service Center, Shanghai, China

**Keywords:** community exercise program, older adults, Qigong, resistance training, type 2 diabetes

## Abstract

**Background:**

Baduanjin and resistance training improve metabolism and function; however, it is unclear whether their combination reduces cardiometabolic risk in older adults with type 2 diabetes mellitus (T2DM). This study aimed to determine the effects of 16-week BDJ combined with resistance training on cardiometabolic risk parameters in older adults with T2DM.

**Methods:**

An assessor-blinded, randomized controlled. A total of 123 participants were enrolled and randomly assigned to either an intervention group (IG) that received a 16-week supervised BDJ combined with resistance training exercise program (2–3 sessions/week, 60 min/session, n=60) or a control group (CG) that received traditional health education without structured exercise (n=63). Participants in the CG were instructed to maintain their usual daily activities and attend monthly health education talks. The primary outcomes were glycosylated hemoglobin (HbA1c) and fasting plasma glucose (FPG) level, body composition, and handgrip strength. The secondary endpoints were blood pressure and lipid levels. Assessments for all outcome measures were performed at baseline and at 4-month follow-up.

**Results:**

Statistically significant group-by-time interactions were observed for weight (B = -1.30, 95% CI [-2.17, -0.44], *p* = 0.003), BMI (B = -0.49, 95% CI [-0.82, -0.17], *p* = 0.003), waist circumference (B = -1.21, 95% CI [-2.09, -0.32], *p* = 0.008), Left handgrip strength (B = 2.02, 95% CI [0.98, 3.05], *p* < 0.001), Right handgrip strength (B = 3.75, 95% CI [2.46, 5.05], *p* < 0.001), FPG (B = -0.81, 95% CI [-1.43, -0.20], *p* = 0.010), HbA1c (B = -0.39, 95% CI [-0.64,-0.14], *p* = 0.003). In contrast, none of the secondary outcome measures showed statistically significant differences between the two groups at the 4-month follow-up.

**Conclusions:**

This study provides evidence that a 16-week program combining BDJ with resistance training can be successfully implemented in a diabetes self-management group for older adults with T2DM. By improving glycemic control, muscle strength, and body composition, the intervention improved selected cardiometabolic risk markers in older adults with T2DM.

**Clinical trial registration:**

http://www.chictr.org.cn, identifier ChiCTR2600123608.

## Background

Older adults have the highest prevalence of type 2 diabetes mellitus (T2DM) (1). Between 2005 and 2050, the number of individuals affected within this demographic is projected to increase by more than 4.5-fold (2). Compared to their non-diabetic counterparts, older adults with T2DM face a markedly higher risk of both acute and chronic complications, including geriatric syndromes, kidney disease, major lower limb amputation, blindness, stroke, and coronary artery disease (3–6). Additionally, they are more likely to require institutional care and experience premature mortality (1, 7, 8). These long-term complications contribute to a diminished quality of life, substantial healthcare costs, and an ongoing rise in T2DM incidence and prevalence (2, 9). Furthermore, older adults with T2DM represent a clinically heterogeneous population (10), and managing diabetes in this group has become a significant clinical and public health challenge. Therefore, Self-management skills are crucial because people with diabetes are responsible for making most of the decisions that influence their diabetes (11). The particularities and challenges of aging that affect diabetes self-management often involve a sense of loss and demand deep readjustment (12).

For most people with T2DM, exercise is recommended for diabetes management and can be undertaken safely and effectively (13). Evidence suggests that for individuals with T2DM, participating in combined training three times per week may offer superior benefits for blood glucose regulation compared with either aerobic or resistance exercise alone (14). Research has demonstrated that resistance training enhances muscular strength and improves glycemic control, as evidenced by reductions in blood glucose and HbA1c levels (15). Similarly, aerobic exercise enhances cardiorespiratory fitness and contributes to improved blood glucose and HbA1c control in patients with T2DM (16, 17). Furthermore, a recent study specifically recommended that older adults with T2DM engage in low-to-moderate-intensity exercise combined with resistance training to mitigate age-related muscle decline (18). However, for many older adults, functional limitations, comorbidities, and lack of motivation can hinder adherence to conventional exercise regimens (10). Therefore, exercise programs designed specifically for this population must be evaluated for their safety, acceptability, and feasibility. Accordingly, developing innovative strategies for diabetes management is of paramount importance.

Baduanjin (BDJ), also termed the Eight-Section Brocades, is a traditional Chinese Qigong practice characterized by eight gentle postures integrated with meditation and controlled respiration, rendering it a low-impact exercise (19, 20). Owing to its uncomplicated and readily mastered routine, BDJ is especially appropriate for older adults with type 2 diabetes (21–23). A recent systematic review and meta-analysis has demonstrated that BDJ enhances physical performance, gait ability, and postural control in Chinese individuals aged 65 years and older (24). Resistance exercise, synonymous with strength training, involves movements using free weights, weight machines, body weight exercises, or elastic resistance bands. Because of the increased prevalence of T2DM with aging, coupled with age-related decline in muscle mass, known as sarcopenia, resistance training can provide additional health benefits to older adults (25). Previous studies have indicated that this type of exercise intervention may benefit older adults with type 2 diabetes. Conventional aerobic exercise and gym-based resistance training often require higher cardiorespiratory demand, complex movement patterns, and access to equipment or facilities, creating substantial barriers for this population (13, 26). In contrast, BDJ combined with elastic-band resistance training, characterized by low-impact movements, adjustable intensity, minimal equipment requirements, and suitability for home- or community-based settings, may be more acceptable and feasible for older adults with T2DM, thereby improving exercise adaptability and adherence (27, 28).

A recent investigation confirmed that combining BDJ with resistance training is safe and feasible for community-dwelling older adults with type 2 diabetes (29). This study provides a feasible intervention strategy for this purpose. However, owing to the lack of data comparisons before and after the intervention, it is currently impossible to determine the feasibility of the exercise intervention plan carried out by community hospital doctors and sports colleges in establishing a diabetes self-management group (30). To date, no randomized controlled trials have evaluated the effects of BDJ combined with resistance training in older adults with type 2 diabetes. Accordingly, the present study sought to evaluate the effectiveness and practical implementation of a 16-week BDJ-plus-resistance exercise program in this population. An innovative diabetes management approach was established, which involved a diabetes self-management group composed of doctors from community hospitals and athletes from sports universities; who conducted exercises such as BDJ and resistance training with elastic bands.

## Methods

### Study design

The present study employed a stratified, parallel-group, assessor-masked randomized controlled trial design, which was implemented at eight community activity centers from July 2025 through October 2025. (Clinical Trials. gov Identifier: ChiCTR2600123608). Randomization was performed at the individual participant level. The study’s reporting adhered to the CONSORT (Consolidated Standards of Reporting Trials) statement (31).

### Participants

This study was conducted in Baoshan, an urban district in Shanghai, China. Eight residential communities were selected from the district using a simple random sampling method. Older adults who met the eligibility criteria were invited to participate in the study. The intervention lasted for 16 weeks (three sessions per week) from July 14 to October 31, 2025. The inclusion criteria required participants to: (1) were aged ≥ 60 years; (2) met the diagnostic criteria of fasting plasma glucose ≥ 7.0 mmol/L, 2-h postprandial plasma glucose ≥ 11.1 mmol/L, and glycated hemoglobin A1c ≥ 6.5% (32); (3) had no significant ketosis or severe cardiovascular disease; (4) had normal communication abilities; (5) provided informed consent and voluntarily participated; and (6) had negative responses to all 10 items on the Physical Activity Readiness Questionnaire (PAR-Q). The exclusion criteria were as follows: (1) concurrent participation in other interventional studies; (2) consistent engagement in moderate-to-vigorous intensity physical activity; (3) type 1 diabetes mellitus or uncontrolled hypertension (systolic blood pressure > 150 mmHg or diastolic blood pressure > 95 mmHg); (4) fasting blood glucose > 16.7 mmol/L; (5) a history of exercise-induced hypoglycemia, diabetic ketoacidosis, elevated ketone bodies (> 4 mmol/L), or microalbuminuria (> 200 mg/L); (6) significant cardiovascular complications, including severe arrhythmia, cardiac insufficiency, coronary heart disease, heart failure, transient ischemic attack, cardiac surgery, myocardial infarction, or angina pectoris at rest or during exercise; (7) musculoskeletal disorders impairing the ability to perform semi-squats or single-leg stands, or the presence of unhealed significant trauma; (8) psychiatric, consciousness, or communication disorders; and (9) Stage III diabetic retinopathy.

### Recruitment, randomization, blinding, and treatment allocation

**Figure 1:**
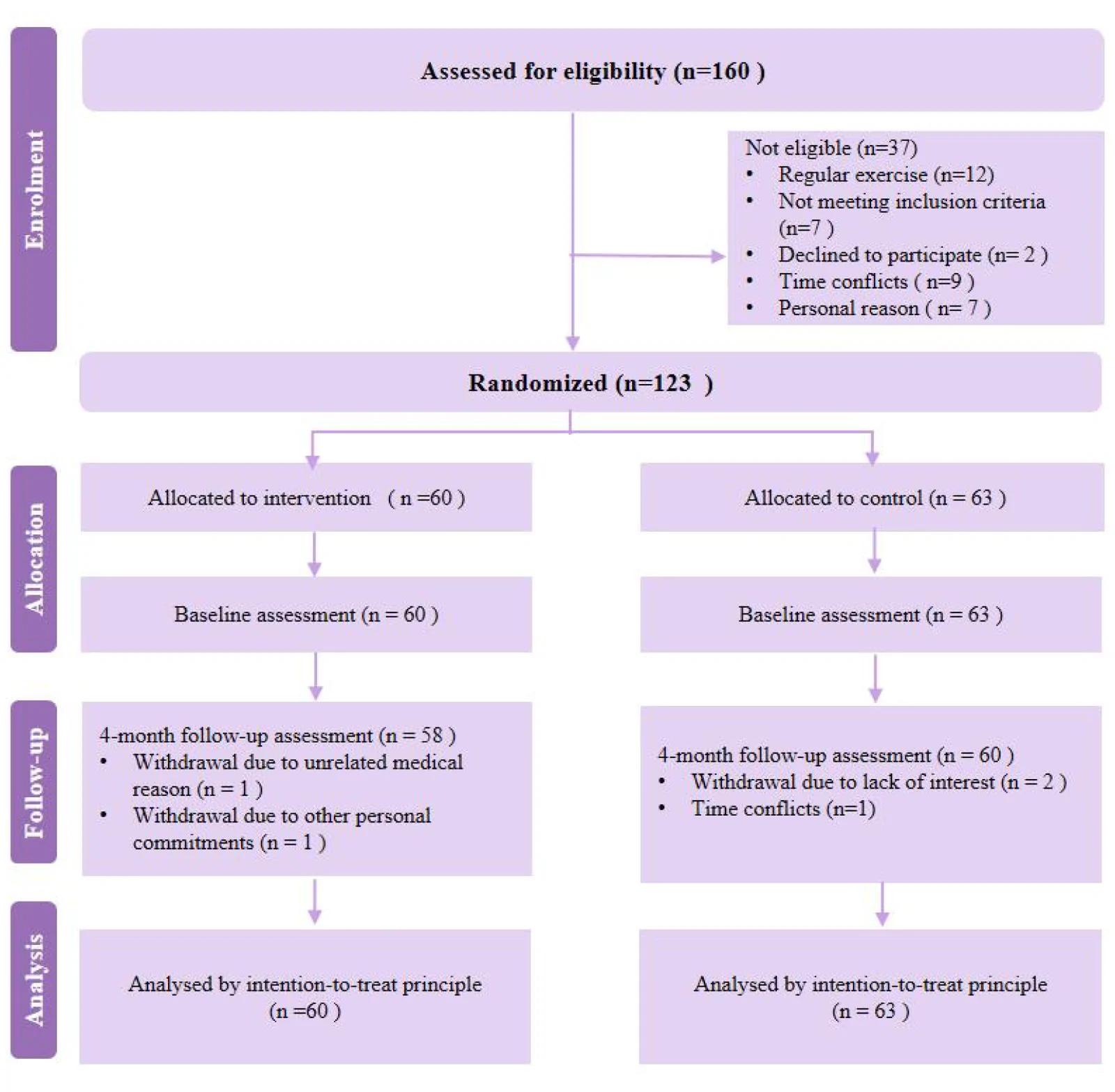
Overview of the recruitment and study timeline.

A total of 160 older adults were recruited from eight communities in Baoshan District, Shanghai. Following baseline assessments, each eligible participant was randomly allocated in a 1:1 ratio to either an intervention group (BDJ combined with resistance training) or a control group (no exercise). A third party generated the individual random allocation sequence using Research Randomizer (https://www.randomizer.org/), which assigned numeric codes (1 = intervention, 2 = control) and sealed these codes in opaque, sequentially numbered envelopes. After enrollment, the research assistants opened the envelopes to reveal each participant’s group allocation. For practical implementation and to minimize cross-group contamination, participants in the same group were arranged to attend designated community activity centers for their respective interventions. These centers were used only as intervention delivery venues and were not involved in the randomization process. The recruitment personnel and trial statistician were blinded to the group assignments. Participants were informed that the study involved a sports-based intervention but were not aware of their specific group allocations. The exercise trainer (first author) could not be blinded to the allocation; however, he remained unaware of the study hypotheses and was provided only with the names of the participants in the intervention group. Two WeChat groups were established to facilitate communication and to foster trust. **Figure 1** presents an overview of the recruitment and study timelines.

### Intervention process

A community-based self-management group for patients with type 2 diabetes was established through collaboration between a community health center and sports school. The intervention provided participants with scientific monitoring of blood glucose and blood pressure, coupled with structured exercise interventions conducted by professional trainers. Additionally, the intervention group encouraged peer sharing and discussions on exercise and dietary management among participants.

Both the intervention and control groups were provided with the same health education content. The intervention group underwent a combined exercise intervention delivered through a community-based self-management group established by a sports university and a cooperating hospital. Before each session, blood pressure and glucose levels were monitored by the physicians. The exercise intervention lasted 60 min and was conducted under the guidance of a professional coach and supervised by a medical doctor. Participants recorded their exercise completion in an exercise diary, received weekly telephone follow-ups, and were reminded to log their dietary intake using a WeChat group.

The control group received routine diabetes care in accordance with the National Essential Public Health Service Specifications (2022 Edition) (32); without any additional structured exercise intervention; and maintained their usual lifestyle habits. The routine care included: (1) quarterly follow-ups comprising measurements of blood pressure, blood glucose, and body weight, along with lifestyle counseling; and (2) health education and medication guidance. **Supplementary Material II** gives details of the contents of regular health management.

All participants in both groups received an equal honorarium upon completion of the intervention as a token of their appreciation. The trial protocol is summarized in **Table 1**.

**Table 1:** Summary of the intervention protocol.

		Intervention group (n= 60)	Control group (n= 63)
Study population Intervention content		older patients with type 2 diabetes (1) Before each exercise: Blood pressure and blood sugar monitoring. (2) Combined Exercise Program: warm-up(6mins), Baduanjin (24 mins), Elastic-band resistance training (24mins), cooldown(6mins). (3) Peer Support: Facilitated via a dedicated WeChat group for sharing and reminders. (4) Regular health education	older patients with type 2 diabetes (1) Medication guidance and adherence counseling. (2) Quarterly follow-ups comprising measurements of blood pressure, fasting plasma glucose, and body weight. (3) Lifestyle counseling (diet, physical activity) as per national guidelines.
Intervention frequency and time	Weeks 1-4 Weeks 5-16	Every Tuesday and Friday at 8:00am. lasting 60 minutes each, for four consecutive weeks. Every Monday, Wednesday and Friday at 8:00am. lasting 60 minutes each, for twelve consecutive weeks.	60-minute health education talk and follow-up one service every 4 weeks (4 sessions total).
Instruments		(1) Socio-demographic questionnaire (2) Collecting fasting blood (3) Body composition test (4) Handgrip strength test (5) blood pressure measuring	(1) Socio-demographic questionnaire (2) Collecting fasting blood (3) Body composition test (4) Handgrip strength test (5) blood pressure measuring

### Intervention group

Through diabetes self-management groups established in partnership between community health centers and a sports school, the IG completed a 16-week structured exercise regimen. The program consisted of BDJ practice combined with resistance band training. A total of 44 supervised sessions, each lasting 60 min, were conducted in the community activity rooms. All 44 sessions were conducted by certified instructors following a standardized manual, with random spot-checks performed by senior researchers to ensure protocol consistency. The health education portion of each session lasted for 30 min and focused on glucose monitoring, diet, and self-management. The exercise structure of each session was as follows: a 6-minute warm-up, 24 minutes of BDJ instruction (comprising two 12-minute sets), 24 minutes of resistance band training, and a 6-minute cooldown. **Supplementary Material II** provides details of the exercise content. During the initial four weeks, the participants engaged in two training sessions per week, concentrating on mastering the fundamental BDJ and resistance band techniques. In the subsequent 12 weeks, the frequency of sessions increased to three per week, during which participants were required to execute the complete BDJ routine alongside the full resistance training protocol. To support adherence and home practice, all participants received a printed illustrated manual and access to an online instructional video. They were also encouraged to log their extra practice sessions and share their progress in a dedicated WeChat group for reinforcement and for peer support. To ensure safety, a designated healthcare professional monitored the participants during each session. Participants’ blood pressure, blood glucose, arterial blood oxygen saturation, and heart rates were measured before and after each session. Participant training was terminated immediately if any of the following safety criteria were met: (1) Hypertension is characterized by a systolic blood pressure of ≥ 130 mmHg or a diastolic blood pressure of ≥ 80 mmHg. (2) Fasting blood glucose>16.7mmol/L, there have been multiple episodes of hypoglycemia, or the blood glucose level fluctuates greatly; (3) Oxygen saturation falling below 95%; (4) Excessively high heart rate (≥ 90 beats per minute); (5) Experience of dizziness or visual disturbances; (6) Report of any other discomfort.

### Control group

Throughout the 16-week study period, participants assigned to the CG participated in a 60-minute health education session every four weeks in addition to receiving standard diabetes follow-up services. The educational topics addressed included blood glucose monitoring, maintenance of physical function and psychological well-being, and prevention of age-related health risks and diseases. They were instructed to maintain their usual lifestyle without engaging in any additional physical exercise and to adhere to their pre-existing dietary and medication regimens (including drug types and dosages). Diabetes follow-up services included: (1) follow-up assessments, comprising measurements of blood pressure, fasting plasma glucose (FPG), and body weight, as well as inquiries regarding disease status, lifestyle, and medication use; (2) stratified interventions, which involved medication guidance, adjustment of drug regimens, referrals when necessary, and health education; and (3) health examinations, consisting of routine physical examinations as well as checks of oral health, vision, hearing, and motor function (32).

### Instruments

Outcomes were evaluated at baseline and at the 4-month follow-up by trained assessors blinded to group allocation.

### Socio-demographic questionnaire

A locally developed questionnaire was used to collect sociodemographic data from older participants. The data gathered included variables such as age, sex, marital status, educational attainment, presence of chronic conditions, annual household income, self-care capacity, blood glucose management strategies, smoking and alcohol consumption habits, and the frequency of physical exercise.

### Primary outcome measures

#### Sugar metabolism outcome measures

Participants were instructed to fast for at least 8 hours (water permitted) prior to venous blood sampling (33). Fasting blood glucose (FBG) levels were measured using an automated clinical chemistry analyzer (Hitachi 7600-020, Hitachi High-Tech Corporation, Tokyo, Japan) (34). Glycated hemoglobin (HbA1c) was measured using a high-performance liquid chromatography (HPLC) system (D-10™ Hemoglobin Testing System, Bio-Rad Laboratories, Hercules, CA, USA) (35).

#### Body composition

Height, body weight, body mass index (BMI), and body composition were assessed using a bioelectrical impedance analyzer (InBody 770, InBody Co., Ltd., Seoul, South Korea) (36).

#### Functional outcome

Handgrip strength was measured using a digital handgrip dynamometer (T.K.K.5401; Takei Scientific Instruments Co., Ltd., Niigata, Japan). The participants were seated upright with their shoulders adducted and neutrally rotated, elbow flexed at approximately 90°, forearm in a neutral position, and wrist in a neutral position (37).

### Secondary cardiometabolic outcomes

#### Lipid metabolism outcome measures

Total cholesterol (TC), triglyceride (TG), high-density lipoprotein cholesterol (HDL-C) 、low-density lipoprotein cholesterol (LDL-C), and very low density lipoprotein(VLDL) levels were measured using an automated clinical chemistry analyzer (Hitachi 7600-020, Hitachi High-Tech Corporation, Tokyo, Japan) (34).

#### Blood pressure

Systolic blood pressure (SBP) and diastolic blood pressure (DBP) were measured using a validated upper-arm-type fully automatic electronic blood pressure monitor (HEM-907, Omron Healthcare Co., Ltd., Kyoto, Japan) (38). using standardized office blood pressure measurement procedures (39).

#### Sample size calculation

The sample size was determined *a priori* based on the primary outcome, change in HbA1c (%) from baseline to 16 weeks. An analysis of covariance (ANCOVA) model adjusting for baseline HbA1c was assumed (40). A between-group difference of 0.5% was prespecified as clinically meaningful and consistent with prior randomized controlled trials of Tai Chi in adults with type 2 diabetes (41, 42). The standard deviation of HbA1c at follow-up was assumed to be 1.2%, and the correlation between baseline and follow-up HbA1c was assumed to be 0.75 (43, 44). With a two-sided α of 0.05 and 80% power, the required sample size was approximately 40 participants per group. Considering an anticipated attrition rate of 20%, we aimed to recruit 50 participants per group (total N = 100).

### Data collection and statistical analysis

Data were collected by research assistants who were blinded to group allocation. Participants completed face-to-face questionnaires at baseline and one week after the intervention. On the morning of blood collection, participants arrived at the community center activity room at 07:30 after a 12-hour overnight fast. Venous blood samples were drawn from the antecubital vein by trained medical staff. These samples were subsequently used for biochemical analysis, with a series of biochemical indicators measured by the community health center laboratory.

Prior to the main analysis, missing data were handled using multiple imputations (MI) under the missing-at-random (MAR) assumption. The dataset was structured in a long format (two records per participant: baseline and 4-month follow-up), yielding 246 observations. The overall missing proportion was 2.65%, with missing data concentrated in several behavioral variables, while the primary and secondary outcome measures were nearly complete. MI was performed using the mice package (Multivariate Imputation by Chained Equations) in R software (version 4.x) (45). The fully conditional specification (FCS) approach based on chained equations was applied for iterative imputation of each incomplete variable. Predictive mean matching (PMM) was selected as the imputation method (46), which is robust to non-normal distributions of continuous variables and ensures that imputed values remain within the plausible range of observed data. The following imputation parameters were specified: 10 independent imputed datasets (m = 10), 50 iterations per dataset (maxit = 50), and a random seed of 2024 for reproducibility. The predictor matrix included all available outcome and covariate variables, with the exception of participant ID, to preserve the missing-at-random assumption.

Data were analyzed using R version 4.2.0 (R Foundation for Statistical Computing, Vienna, Austria). Continuous variables are described as mean ± standard deviation, and categorical variables are reported as counts and percentages. Group comparisons at baseline were performed using independent-samples t-tests for continuous variables and chi-square tests for categorical variables. To evaluate longitudinal changes in outcomes and account for intra-individual correlations across repeated measurements, linear mixed-effects models were fitted separately to each of the 10 imputed datasets, and the results were pooled according to Rubin’s rules (47). In these models, time (baseline vs. post-intervention), group (intervention vs. control), and their interaction were defined as fixed effects, with age and sex included as pre-specified covariates. A random intercept for each participant was included to model within-subject variability. Because randomization was performed at the individual level, the community centers where the interventions took place were not treated as clustering units in the primary analysis. Model assumptions—specifically, normality of residuals and homoscedasticity—were visually inspected using Q-Q plots and residual-versus-fitted plots and were found to be adequately satisfied. Consistent with the intention-to-treat principle, all 123 randomized participants were included in the analysis. Model estimates are reported as regression coefficients (β) with corresponding 95% confidence intervals and P-values.

Although multiple primary outcomes (glycemic control, body composition, and muscle strength) were evaluated, they represent distinct physiological domains and were not subjected to strict multiple comparison adjustments (e.g., Bonferroni correction), as such adjustment could inflate Type II error rates and obscure potentially meaningful clinical signals in this exploratory community-based trial. Nevertheless, the secondary cardiometabolic outcomes (blood pressure and lipid profile) should be interpreted as exploratory, and the findings warrant cautious interpretation. Two-sided P-values < 0.05 were considered statistically significant.

### Ethical considerations

This study was approved by the Ethics Committee of Luodian Hospital, Baoshan District, Shanghai (IRB Ref. No.: LDYY-KY-2024-23). All participants received comprehensive information regarding the study, and written informed consent was obtained from each individual prior to the initiation of data collection. Furthermore, the confidentiality of the questionnaire responses was stringently maintained; no data were disclosed to any third party without the explicit consent of the participants.

## Results

### Demographics

Eight batches of elderly T2DM participants were recruited through the community health service center with eight community partner senior activity center. Therefore, 123 participants were recruited and randomly assigned to the intervention (n = 60) or control group (n = 63). Of the 160 older adults referred to the study, 12 reported engaging in regular exercise, seven did not meet the inclusion criteria, two declined to participate, nine were excluded because of scheduling conflicts, and seven withdrew for personal reasons. In the IG, one participant withdrew due to unrelated medical reasons and another due to other personal commitments. In the CG, two participants withdrew due to lack of interest, and one due to scheduling conflicts. A total of 123 participants with available baseline data were included in the final analysis. The pre-randomization exclusion rate was 23.1%. This was primarily due to the failure to meet the eligibility criteria or safety considerations. After randomization, the loss-to-follow-up rate at 4 months was 4.1%. A flowchart of the recruitment process and specific reasons for participant dropout are shown in **Figure 1**. There was no significant difference in the baseline data between the two groups (P > 0.05); the groups were balanced and comparable, as shown in **Table 2**.

**Table 2:** Baseline data comparison results between the intervention group and the control group.

		Intervention group(n=60)	Control group(n=63)	P-value
		N	N	
Gender(n(%))	Male	28(46.7)	38(60.3)	0.566
	Female	32(53.7)	25(39.7)	
Age(years)	60-69	65.60(2.41)	65.35(2.42)	0.181
Marital status(n(%))	Unmarried	1(1.7)	0(0.0)	0.524
	Married	55(91.7)	61(96.8)	
	No spouse	3(5.0)	1(1.6)	
	Divorced	1(1.7)	1(1.6)	
Education(n(%))	undergraduate college	1(1.7)	0(0.0)	<0.001
	junior college	4(6.7)	7(11.1)	
	Vocational school	14(23.3)	2(3.2)	
	High school	16(26.7)	36(57.1)	
	Middle school	18(30.0)	15(23.8)	
	primary school	7(11.7)	0(0.0)	
annual income(n(%))	<10000	1(1.7)	0(0.0)	0.643
	10000-29999	4(6.7)	2(3.2)	
	30000-49999	13(31.7)	12(19.0)	
	50000-99999	20(33.3)	26(41.3)	
	100000-1499999	7(11.7)	11(17.5)	
	150000-1999999	12(20.0)	8(12.7)	
	≥200000	3(5.0)	4(6.3)	
Smoking(n(%))	Never	34(56.7)	33(52.4)	0.180
	quit smoking	4(6.7)	11(17.5)	
	smoking	22(36.7)	19(30.2)	
Drinking(n(%))	Never	44(73.3)	43(68.3)	0.238
	occasionally	7(11.7)	14(22.2)	
	often	9(15.0)	6(9.5)	
Height(cm)		163.55(8.99)	166.81(9.48)	0.053
Weight		66.06(9.92)	68.70(12.40)	0.197
BMI		24.66(3.00)	24.70(4.15)	0.961
WC		86.58(8.29)	86.36(10.27)	0.894
HC		94.65 (5.57)	93.50 (6.81)	0.306

Date presented in n (%) or mean (SD).

### Comparisons of outcome variables of older adults with T2DM

**Figure 2:**
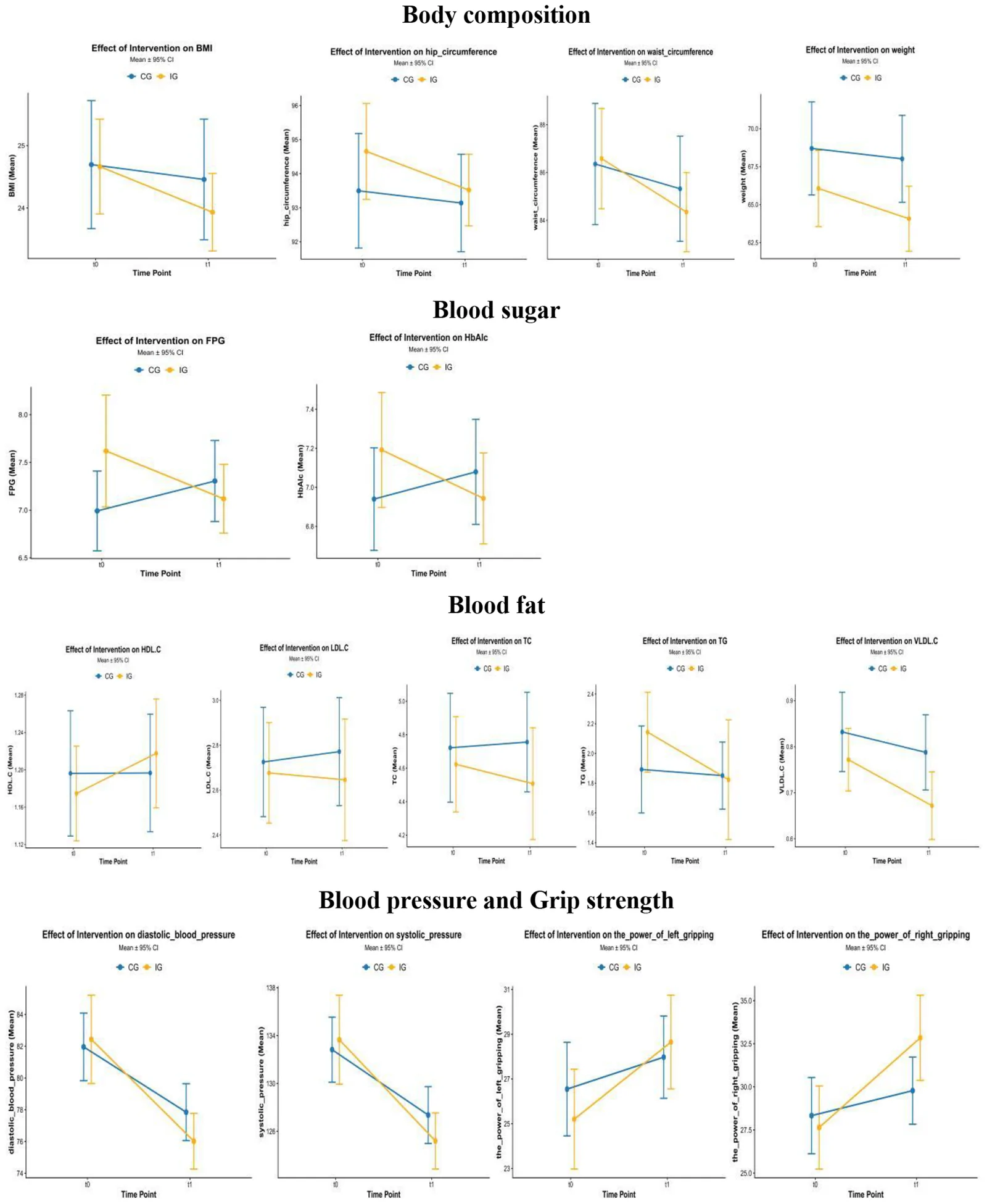
Profile plots of interaction effects before and after the intervention of cardiac metabolic risk parameters.

**Table 3** and **Supplementary Material III** show the comparison of outcome variables of older adults between the intervention and control groups at baseline and 4-month follow-up. **Figure 2** displays the profile plots of the interaction effects over time.

**Table 3:** Comparison of outcome variables of older adults between intervention and control groups at different time points.

	Group (n)	Assessment		Group effect	Time effect	Group by time interaction effect		
		T0(mean ± SD)	T1(Mean ± SD)			Estimate(95%CI)	S.E	P
Primary outcomes								
weight	Intervention(60)	66.06 ± 9.92	64.07 ± 8.45	0.437	<0.001*	-1.3(-2.17, -0.44)	0.44	0.003*
	Control(63)	68.70 ± 12.40	68.01 ± 11.60					
BMI	Intervention(60)	24.66 ± 3.00	23.93 ± 2.46	0.555	<0.001*	-0.49(-0.82, -0.17)	0.16	0.003*
	Control(63)	24.70 ± 4.15	24.46 ± 3.91					
WC	Intervention(60)	86.58 ± 8.29	84.34 ± 6.54	0.498	<0.001*	-1.21(-2.09, -0.32)	0.45	0.008*
	Control(63)	86.36 ± 10.27	85.32 ± 8.91					
HC	Intervention(60)	94.65 ± 5.57	93.52 ± 4.15	0.291	<0.001*	-0.78(-1.70, 0.14)	0.46	0.097
	Control(63)	93.50 ± 6.81	93.14 ± 5.79					
FPG	Intervention(60)	7.62 ± 2.31	7.12 ± 1.42	0.173	0.026*	-0.81(-1.43, -0.20)	0.31	0.010*
	Control(63)	6.99 ± 1.69	7.31 ± 1.72					
HbA1c	Intervention(60)	7.19 ± 1.16	6.94 ± 0.92	0.631	0.007*	-0.39(-0.64, -0.14)	0.13	0.003*
	Control(63)	6.94 ± 1.06	7.08 ± 1.09					
LHGS	Intervention(60)	25.20 ± 8.83	28.65 ± 8.29	0.872	<0.001*	2.02(0.98, 3.05)	0.52	<0.001*
	Control(63)	26.55 ± 8.48	27.97 ± 7.44					
RHGS	Intervention(60)	27.64 ± 9.52	32.84 ± 9.77	0.535	<0.001*	3.75(2.46, 5.05)	0.65	<0.001*
	Control(63)	28.33 ± 8.96	29.78 ± 7.88					
Secondary outcomes								
SBP	Intervention(60)	133.66 ± 14.70	125.19 ± 9.29	0.731	<0.001*	-3.00(-6.46, 0.46)	1.75	0.089
	Control(63)	132.83 ± 11.02	127.36 ± 9.60					
DBP	Intervention(60)	82.43 ± 11.00	76.02 ± 6.93	0.857	<0.001*	-2.31(-4.91, 0.30)	1.31	0.082
	Control(63)	81.96 ± 8.62	77.85 ± 7.26					
TC	Intervention(60)	4.62 ± 1.13	4.51 ± 1.32	0.119	0.447	-0.15(-0.57, 0.27)	0.21	0.482
	Control(63)	4.72 ± 1.32	4.76 ± 1.21					
TG	Intervention(60)	2.14 ± 1.06	1.82 ± 1.59	0.246	0.037*	-0.28(-0.70, 0.14)	0.21	0.190
	Control(63)	1.89 ± 1.18	1.85 ± 0.91					
HDL-C	Intervention(60)	1.17 ± 0.20	1.22 ± 0.23	0.053	0.159	0.04(-0.04,0.13)	0.04	0.318
	Control(63)	1.20 ± 0.27	1.20 ± 0.25					
LDL-C	Intervention(60)	2.68 ± 0.89	2.65 ± 1.07	0.277	0.813	-0.08(-0.44,0.28)	0.18	0.673
	Control(63)	2.73 ± 0.99	2.77 ± 0.97					
VLDL-C	Intervention(60)	0.77 ± 0.27	0.67 ± 0.29	0.091	0.035*	-0.06(-0.19,0.07)	0.07	0.400
	Control(63)	0.83 ± 0.35	0.79 ± 0.33					

Data are presented as estimated mean (standard deviation), baseline covaried. *Statistical significance, BMI body mass index, WC waist circumference, HC hip circumference, FPG fasting blood glucose, HbA1c glycosylated hemoglobin, LHGS left handgrip strength, RHGS right handgrip strength, SBP systolic blood pressure, DBP diastolic blood pressure, TC total cholesterol, TG triglyceride, HDL-C high-density lipoprotein cholesterol, LDL-C low-density lipoprotein cholesterol, VLDL-C very low-density lipoprotein cholesterol.

For the primary outcomes, significant group-by-time interactions were observed for weight (B = -1.30, 95% confidence interval (CI) [-2.17, -0.44], *p* = 0.003), BMI (B = -0.49, 95% CI [-0.82, -0.17], *p* = 0.003), WC (B = -1.21, 95% CI [-2.09, -0.32], *p* = 0.008), LHGS (B = 2.02, 95% CI [0.98, 3.05], *p* < 0.001), RHGS (B = 3.75, 95% CI [2.46, 5.05], *p* < 0.001), FPG (B = -0.81, 95% CI [-1.43, -0.20], *p* = 0.010), HbA1c (B = -0.39, 95% CI [-0.64, -0.14], *p* = 0.003), In contrast, no significant group-by-time interaction was detected for hip circumference (B = -0.78, 95% CI [-1.70, 0.14], *p* = 0.097).

For the secondary outcomes, none of the group × time interaction effects reached statistical significance: SBP (B = -3.00, 95% CI [-6.46, 0.46], *p* = 0.089), DBP (B = -2.31, 95% CI [-4.91, 0.30], *p* = 0.082), TC (B = -0.15, 95% CI [-0.57, 0.27], *p* = 0.482), TG (B = -0.28, 95% CI [-0.70, 0.14], *p* = 0.190), HDL-C (B = 0.04, 95% CI [-0.04, 0.13], *p* = 0.318), LDL-C (B = -0.08, 95% CI [-0.44, 0.28],*p* = 0.673), VLDL-C (B = -0.06, 95% CI [-0.19, 0.07], *p* = 0.40), indicating that there were no between group differences in the changes in these outcomes over time between the intervention and control groups (**Table 3**, **Figure 2**).

Post-hoc pairwise comparisons revealed statistically significant within-group improvements from baseline to the 4-month follow-up in the IG for body composition, glycemic measures, blood pressure, and handgrip strength. For example, weight decreased by 1.99 kg (95% CI [1.38, 2.60], *p* < 0.001). In the CG, no significant within-group changes were observed for body composition, glycemic parameters, or lipid profiles.

### Safety outcomes

During the 16-week intervention period, no exercise-related adverse events were reported in any of the 60 participants in the intervention group. Specifically, no participant experienced hypoglycemic episodes, symptomatic hypotension, syncope, chest pain, palpitations, or musculoskeletal injuries that required medical attention. Importantly, none of the supervised sessions were terminated prematurely based on the pre-specified safety criteria (i.e., blood pressure ≥130/80 mmHg, fasting glucose >16.7 mmol/L, oxygen saturation <95%, heart rate ≥90 bpm, dizziness, or visual disturbance). These findings confirm that the combined BDJ and resistance training program, when delivered under medical supervision, is safe for community-dwelling older adults with T2DM.

### Compliance

The mean attendance rate across all 44 supervised sessions was 90.6%. Of the 60 participants in the intervention group, 52 (86.7%) attended at least 90% of the sessions, with 3 (5.0%) achieving perfect attendance; and 5 (8.3%) maintaining a 70% attendance rate. Although initial adherence was suboptimal, the introduction of daily reminders, free health monitoring, and token rewards was associated with gradual improvement throughout the study period. Participant diaries documented that the primary reasons for missed sessions were travel, temporary illness, and scheduling conflicts that arose.

## Discussion

Based on a diabetes self-management group, the combined BDJ and resistance training program significantly improved key cardio-metabolic risk factors in older adults with type 2 diabetes compared to routine health management alone. Participants in the intervention group demonstrated statistically and clinically significant reductions in body weight, body mass index, waist circumference, fasting plasma glucose, and glycated hemoglobin levels, along with; a significant increase in bilateral handgrip strength. However, no significant between-group differences were observed in blood pressure or lipid profile during the study period. Interestingly, we found that after the intervention, body weight, BMI, waist circumference, handgrip strength, and blood pressure exhibited certain degrees of improvement in the control group, although the group × time interaction effects were not statistically significant. We reasonably and optimistically conjectured that the intervention in the control group would also be beneficial in improving body weight, BMI, waist circumference, handgrip strength, and blood pressure in the control group. However, routine health management appeared to have a limited effect on improving FPG and HbA1c levels in this study. These findings suggest that a low-to-moderate intensity, multimodal exercise program integrating traditional mind–body exercise with resistance training is an effective and feasible strategy for improving key cardiometabolic risk factors in older adults with T2DM at the community level. In diabetes management, our intervention has two contributions to modern-day studies on cardiometabolic risk factors in older adults with T2DM. First, in previous research on exercise interventions to prevent and control Cardiometabolic Risk in patients with type 2 diabetes, few studies have investigated the combined application of BDJ with resistance training, particularly in older adult populations, and only investigated the effects of individual aerobic or resistance training modalities (48–50). Moreover, our study offers important insights into the feasibility and effectiveness of delivering multimodal exercise interventions in community settings, which is crucial for increasing accessibility and adherence in older adults with T2DM. Many previous studies have focused on clinic-based or hospital-centered interventions that are less accessible to older adults living in the community than home-based interventions (18, 51).

Some studies have shown that when FPG exceeds 5.6 mmol/L, the related mortality risk begins to increase (52). Further studies have found that a higher mortality risk occurs for FPG of 6.1–6.9 mmol/L (53). Therefore, effective strategies to reduce FPG levels should be actively explored. Our results showed that BDJ with resistance training significantly alleviated the abnormal FPG levels of patients with T2DM in a relatively short time (approximately 4 months). The improvements in glycemic control observed in this study can be attributed to several underlying physiological mechanisms. Resistance training, a key component of this intervention, promotes an increase in muscle mass and enhances insulin sensitivity. As muscle mass declines with age, older adults with T2DM often experience increased insulin resistance, contributing to poor glycemic control. Resistance training stimulates glucose uptake into muscle cells by increasing the ability of insulin to stimulate glucose transporter type 4 (GLUT4) in muscle cells (54). By enhancing muscle mass and improving insulin sensitivity, resistance training can help mitigate insulin resistance and improve glucose metabolism in older adults with T2DM. This is consistent with a recent meta-analysis that examined the effects of RT in adults with T2DM (50, 55), which showed that resistance training significantly improved HbA1c and FPG levels in T2DM patients compared to non-exercise controls. In addition to resistance training, BDJ exercise improves mitochondrial function in muscle cells, enhances glucose oxidation, and reduces reliance on fat as an energy source. Mitochondria play a critical role in energy metabolism, and improved mitochondrial function allows muscles to utilize glucose more efficiently, leading to better blood glucose regulation (56, 57). The slow, deliberate movements and controlled breathing of BDJ likely stimulate the parasympathetic nervous system, reducing the chronic sympathetic activation often seen in T2DM, which further supports insulin sensitivity and glucose regulation.

WC is a sensitive measure of abdominal obesity that reflects the degree of fat accumulation in the abdominal cavity and the viscera. WC > 85 cm is closely related to the incidence of diabetes and cardiovascular complications (58). For every 1-cm increase in WC, the risk of CVD increased by 3.2% (59). Visceral fat contributes significantly to insulin resistance by releasing inflammatory cytokines and free fatty acids. The reduction in waist circumference and BMI in the intervention group suggests that the combined exercise regimen helped reduce visceral fat (58), thereby alleviating the inflammatory burden on insulin-sensitive tissues. This reduction in visceral fat plays a crucial role in improving glycemic control and reducing the risk of metabolic complications in T2DM patients (60, 61), as demonstrated in several previous studies (62). In this study, most participants had abdominal obesity, but after 4-month of BDJ and resistance training or regular health management, the WC of participants in the IG and CG groups decreased by 2.6% and 1.2%, respectively, and weight decreased by 3% and 1%. Importantly, although both groups demonstrated within-group improvements, the statistically significant group-by-time interactions for weight, BMI, and waist circumference suggested that the improvements were more pronounced in the IG. The modest reductions observed in the control group are plausible in the context of routine health management and repeated assessments, which may increase participants’ health awareness and prompt self-initiated behavioral adjustments (e.g., diet or daily activity) even without structured exercise training. Nevertheless, the larger proportional reductions in central adiposity and body weight in the intervention group are clinically relevant, as central adiposity is closely linked to insulin resistance and cardiometabolic risk in older adults with T2DM. In addition, while a 5–10% weight reduction is often highlighted as a threshold for cardiovascular risk reduction (63–65), for individuals with type 2 diabetes who demonstrate inadequate control of glycemic levels, blood pressure, and lipid concentrations, or who exhibit other obesity-related metabolic disorders, a modest yet sustained weight reduction (equivalent to 3–7% of total body weight) can improve glycemic, blood pressure, and lipid profiles and may potentially reduce the necessity for disease-specific pharmacological interventions (58, 66). Therefore, even modest exercise-related reductions in waist circumference and adiposity may be clinically meaningful in older adults with T2DM, as improvements in glycemic control accompanied by a lower visceral fat burden can collectively indicate a favorable shift in cardiometabolic risk (67).

The increase in handgrip strength observed in the intervention group was another critical outcome. Handgrip strength is a clinically meaningful marker in geriatric populations because it correlates with overall muscle function, disability risk, and mortality outcomes, including cardiovascular mortality (60, 68, 69). Resistance training enhances strength primarily via early neuromuscular adaptations, followed by an increase in muscle cross-sectional area and improved muscle quality with sustained training (70). These adaptations are particularly important in older adults with T2DM, who often exhibit accelerated declines in muscle mass and strength, which can exacerbate insulin resistance and functional limitations (71). In parallel, BDJ, with its focus on balance and coordination, further reinforces proprioceptive input, balance control, and movement coordination, thereby supporting neuromuscular efficiency and functional performance. This is consistent with evidence that BDJ improves balance-related outcomes and reduces fall risk in older adults (72–74).

In terms of blood lipid levels and blood pressure, our mixed-effects models did not identify statistically significant group-by-time interactions for SBP, DBP, or lipid parameters (TC, TG, HDL-C, LDL-C, and VLDL-C), indicating no differential change between groups over the 4-month period. A meta-analysis of randomized controlled trials reported that supervised exercise improved blood pressure control, lowered LDL-C, and increased HDL-C levels in adults with diabetes (75). Consistent with this, major position statements for diabetes management emphasize exercise as a core non-pharmacological strategy for improving multiple cardiovascular risk markers, although the magnitude of lipid and blood pressure changes is typically modest and heterogeneous across studies (13). The lack of significant changes in these parameters may be attributed to various factors. First, the baseline levels of blood pressure and lipid measures in our population were relatively well-controlled, which may have limited our ability to detect changes over the short intervention period (76). Second, blood pressure and lipid levels are influenced by various factors beyond exercise, including medication use, dietary patterns, and genetic predisposition, which were not directly addressed by the intervention (13). Finally, compared with outcomes such as glycemia and body composition, detectable between-group differences in blood pressure and lipid profiles may require a longer intervention duration or higher training volume, especially in community-based older populations with concurrent medical management (76, 77).

## Limitation

Several limitations of this study should be acknowledged. First, the intervention duration was relatively short, and the long-term sustainability of the observed benefits is unclear. Second, direct measures of insulin sensitivity, inflammatory markers, muscle mass, and mental health outcomes were not assessed, which limited mechanistic inference. Third, changes in antihypertensive and lipid-lowering medications were not systematically recorded or controlled, which may have confounded the interpretation of blood pressure and lipid levels. Fourth, participants were recruited from a single urban district, which may restrict the generalizability of the results to other populations or settings. Fifth, the intervention and control groups were not matched for contact intensity or attention. Participants in the intervention group received substantially more supervision, monitoring, social support (via WeChat), and behavioral reinforcement than those in the control group, who received only routine care and monthly health education. Therefore, the observed improvements may reflect the combined effects of structured exercise, enhanced self-monitoring, and psychosocial support rather than the specific physiological effects of Baduanjin combined with resistance training alone. Sixth, the fidelity and adherence assessments had notable limitations. For supervised sessions, although delivered by trained instructors using a standardized protocol, we did not conduct formal fidelity checks (e.g., independent observations or video recordings). For home-based practice, adherence was measured solely via self-reported diaries, which are prone to recall and social desirability biases, without objective verification using accelerometers or smart bands. Consequently, the lack of formal fidelity measures limits the accurate assessment of both the true intervention dose and the reliability of adherence data. Future studies should incorporate longer follow-ups, objective body composition and metabolic measurements, systematic medication tracking, multicenter recruitment, and accelerometer-based monitoring to complement self-reports, thereby better elucidating the physiological mechanisms and long-term effects of this combined exercise intervention.

## Conclusion

In conclusion, a 16-week community-based BDJ exercise combined with resistance training significantly improved glycemic control, body composition, and muscle strength in older adults with type 2 diabetes. These benefits are likely mediated by enhanced skeletal muscle glucose uptake, reduced central adiposity, and improved neuromuscular function. This low-cost, culturally adaptable, and physiologically targeted intervention represents a promising strategy for cardiometabolic risk reduction in the growing population of older adults with T2DM.

## Data Availability

The data underlying the findings in this paper are openly and publicly available and can be found here: https://figshare.com/s/dbaa5029756d6b76018f. If you encounter problems accessing the data, please contact the corresponding author.
