# Supplementary material for: Effectiveness of community—based Baduanjin combined with resistance training on cardiometabolic risk factors in older adults with type 2 diabetes: a 16-week randomized controlled trial
Source: Front Endocrinol (Lausanne). 2026 Aug 27;17:1930328. doi: 10.3389/fendo.2026.1930328 (PMC13557981; doi:10.3389/fendo.2026.1930328)
Supplement: Supplementary file 1 [file SupplementaryFile2.docx]

**Combined Baduanjin and Elastic-band Training Improves Cardiometabolic Risk Profile in Older Adults with Type 2 Diabetes: A 16-Week Randomized Controlled Trial**

**Supplementary Material 2**

I. **Study Protocol**
II. **Statistical analysis plan**
III. **Prespecified primary and secondary outcome measures**
I. **Study Protocol**

**Effects of Exercise on Community-older adults with Type 2 Diabetes Mellitus**

**1Research Background**

The aging process of the Chinese population has accelerated. Data from the five most recent national population censuses show that the proportion of people aged ≥ 60 years in China was 7.62%, 8.57%, 10.33%, 13.26%, and 18.70% in 1982, 1990, 2000, 2010, and 2020, respectively [1]. Globally, the number of individuals with diabetes increased from 108 million in 1980 to 463 million in 2019. In China, the prevalence of diabetes rose sharply from 0.67% in 1980 to 10.4% in 2013. By 2019, the number of patients with diabetes in China had increased to 116 million, with a prevalence exceeding 10%, making China the country with the largest diabetes population in the world [2]. According to the latest national survey on diabetes among the elderly, the number of people aged ≥ 65 years with type 2 diabetes mellitus (T2DM) in China is approximately 35.5 million, ranking first in the world and accounting for 25% of the global elderly T2DM population, with this number continuing to show an increasing trend. The clinical manifestations of diabetes often include fatigue, polyphagia, polydipsia, polyuria, weight loss, poor self-management, and depression, which severely affect the normal daily life of patients. Most T2DM patients have various complications, which can easily lead to blindness, heart disease, kidney disease, and nerve damage, seriously impairing their health [2, 3]. The social and economic burden of managing and addressing diabetes has been increasing annually. In 2019, the International Diabetes Federation estimated that the direct health expenditure on diabetes was 760 billion US dollars and is projected to reach 825 billion US dollars by 2030, of which the cost of treating complications will account for more than 50% of the total health expenditure. Therefore, disease prevention should be prioritized [4-6].

Exercise can regulate blood glucose and improve insulin resistance, making it an effective strategy for preventing T2DM [7-9]. Aerobic, resistance, or various combined exercise modalities contribute to improving glucose regulation, insulin sensitivity, oxidative capacity, and relevant metabolic parameters, and thus represent an effective approach for managing and preventing type 2 diabetes [10]. The latest consensus on exercise guidelines for T2DM, jointly issued by the American Diabetes Association and the American College of Sports Medicine, clearly states that combined resistance and aerobic exercise has a significantly better intervention effect on glycemic stability in T2DM patients than either modality alone; therefore, a combined exercise approach is more strongly recommended [11].

**2 Research Objectives and Significance**

**2.1 Research Objectives**

Through a literature review, expert consultation, and a pilot study, this research employed non-medical interventions to investigate the effects of different self-management group models on body composition and related physiological parameters in elderly patients with type 2 diabetes mellitus (T2DM). The specific objectives are as follows: (1) To examine the impact of a community-based health self-management group, established through collaboration between a sports school and a community health service center, on body composition and glycemic/lipid profiles in elderly T2DM patients. This model provides patients with scientific blood glucose and blood pressure monitoring, alongside professionally coached and structured exercise intervention guidance. (2) To clarify the effects of this intervention by comparing the combined exercise group with a routine management group, thereby providing empirical evidence for optimizing community-based exercise management strategies for T2DM and promoting effective health self-management among patients. Ultimately, this study aims to provide a scientific basis for community health management of T2DM and inform the future development of more targeted and feasible self-management protocols for this population.

**2.2 Research Significance**

Theoretical significance: This study analyzed the effects of a combined exercise intervention program on middle-aged and elderly T2DM patients, offering a theoretical reference for effective and safe self-health management in this cohort.

Practical significance: The findings provide community medical institutions and health self-management groups with a scientific, safe, effective, and easily implementable exercise intervention protocol, thereby enhancing the quality and efficacy of community-based diabetes management. Furthermore, the results serve as a decision-making reference for government agencies in formulating chronic disease community prevention strategies and promoting nationwide fitness initiatives in Taiwan.

**3 Research Methods**

**3.1 Participant Recruitment**

Potential participants with T2DM aged ≥ 60 years who met the inclusion criteriawere identified through the health record system of community health service centers. Recruitment was carried out via the following approaches: (1) placement of research recruitment posters on bulletin boards at community health service centers in Baoshan District, Shanghai; and (2) distribution of informational brochures by community staff (members of the collaborative research team) during community health lectures or health education activities. These brochures clearly and comprehensively detailed the study content, eligibility criteria, and enrollment procedures; (3) dissemination of recruitment information through online platforms, such as community WeChat groups. The posted information included a brief study introduction, researcher contact details, registration methods, and an electronic version of the informed consent document for potential participants to review at their convenience; (4) word-of-mouth referral among elderly individuals and their neighbors; and (5) direct telephone contact with eligible participants identified from the health record system of the community health service centers.

**3.2 Inclusion and Exclusion Criteria for Participants**

The inclusion criteria required participants to: (1) were aged ≥ 60 years; (2) met the diagnostic criteria of fasting plasma glucose ≥ 7.0 mmol/L, 2-h postprandial plasma glucose ≥ 11.1 mmol/L, and glycated hemoglobin A1c ≥ 6.5%(32); (3) had no significant ketosis or severe cardiovascular disease; (4) had normal communication abilities; (5) provided informed consent and voluntarily participated; and (6) had negative responses to all 10 items on the Physical Activity Readiness Questionnaire (PAR-Q). The exclusion criteria were as follows: (1) concurrent participation in other interventional studies; (2) consistent engagement in moderate-to-vigorous intensity physical activity; (3) type 1 diabetes mellitus or uncontrolled hypertension (systolic blood pressure > 150 mmHg or diastolic blood pressure > 95 mmHg); (4) fasting blood glucose > 16.7 mmol/L; (5) a history of exercise-induced hypoglycemia, diabetic ketoacidosis, elevated ketone bodies (> 4 mmol/L), or microalbuminuria (> 200 mg/L); (6) significant cardiovascular complications, including severe arrhythmia, cardiac insufficiency, coronary heart disease, heart failure, transient ischemic attack, cardiac surgery, myocardial infarction, or angina pectoris at rest or during exercise; (7) musculoskeletal disorders impairing the ability to perform semi-squats or single-leg stands, or the presence of unhealed significant trauma; (8) psychiatric, consciousness, or communication disorders; and (9) Stage III diabetic retinopathy.

**4. Research Methods**

4.1 Literature Review

Relevant domestic and international literature on type 2 diabetes mellitus (T2DM), chronic diseases in middle‑aged and elderly populations, and glycemic control was retrieved from the CNKI, PubMed, Web of Science, Embase, and Cochrane databases. This review focused on the epidemiology of T2DM, studies on exercise interventions for T2DM, and the effects of various exercise modalities on T2DM‑related outcomes. Particular attention was given to the association between T2DM and the combined intervention of Baduanjin (a traditional Chinese mind‑body exercise) and elastic band resistance training, with the aim of identifying research gaps and providing a theoretical basis for experimental design.

4.2 Questionnaire Survey

A series of standardized scales with established reliability and validity were administered to all participants before and after the intervention to comprehensively evaluate the effects of the combined exercise program on body composition, muscle mass, and other physiological parameters. The scales employed included the following: (1) General Information Questionnaire: Developed by the researchers, this questionnaire collected sociodemographic data (age, marital status, sex, education level, monthly income, etc.) and general health information (height, weight, body mass index, medical history, and medication use). (2) Summary of Diabetes Self-Care Activities (SDSCA): Originally developed by Toobert et al. (2000) and subsequently adapted for the Chinese population by Sun et al., this scale assesses the frequency of self-management behaviors in diet, exercise, blood glucose monitoring, foot care, and smoking. It comprises six dimensions: dietary management (four items), foot care (two items), blood glucose monitoring (two items), exercise management (two items), smoking (one item), and medication adherence (one item). All dimensions were positively scored, except for smoking, which was negatively scored. (3) Exercise Diary: Designed by the researchers according to the study needs, the exercise diary recorded planned and actual exercise sessions. The participants completed a diary to monitor exercise adherence. The completion rate was calculated as (actual sessions/prescribed sessions) × 100%, where an actual session was defined as completing at least eight of all prescribed movements. Adherence was considered high when the completion rates exceeded 85%.

4.3 Experimental Method

4.3.1 Experimental Design

All participants underwent baseline assessments of body composition, blood glucose, and lipid profiles. The intervention group received a 16‑week combined exercise program consisting of Baduanjin and elastic band resistance training, while the control group followed the routine management protocol of a standard health self‑management program. Participants in the control group were instructed to maintain their habitual lifestyle and not engage in additional structured exercise during the intervention period. All participants maintained their usual diet and medication regimens (including drug type and dosage) throughout the study. Following the final exercise session, post-intervention measurements of body composition, blood glucose, blood lipids, and muscle strength were obtained. The independent variable was the exercise intervention modality, and the dependent variables were body composition, muscle strength, and indicators of glucose and lipid metabolism in middle-aged and elderly T2DM populations.

4.3.2 Outcome Measures

On the test days, at 7:30 a.m., two tubes of fasting venous blood were collected using lithium heparin anticoagulant tubes: one for fasting plasma glucose and related assays and the other for additional blood parameters. Anticoagulated blood samples were centrifuged at 3000 rpm for 10 min, and the plasma supernatant was used for analysis. Glucose metabolism indicators were measured using an automated biochemical analyzer. The measured outcomes included body composition, fasting plasma glucose (FPG), glycated hemoglobin (HbA1c), serum total cholesterol (TC), triglycerides (TG), high-density lipoprotein cholesterol (HDL-C), low-density lipoprotein cholesterol (LDL-C), and heart rate (HR). Body composition was assessed using an InBody Mass Analysis device (Korea). FPG, TC, TG, HDL-C, and LDL-C levels were measured using a Hitachi 766 automatic biochemical analyzer. HbA1c levels were determined using Bio-Rad hemoglobin microcolumns. Heart rate was monitored using a MIO heart rate monitor (USA).

4.3.3 Exercise Intervention

The intervention consisted of three exercise sessions per week for 16 weeks, with each session lasting approximately 60 min. Participants in the intervention group received prior instructions to ensure proficiency in the required movements. The specific exercise prescription was as follows:

(1) Exercise duration: Current national and international guidelines recommend that individuals with diabetes engage in comprehensive physical activity, including aerobic exercise (at least 150 min/week of moderate-to-vigorous aerobic activity) and resistance training (at least two sessions/week, each ≥60 min). The combined exercise program in this study included aerobic exercise (repetitive, continuous movements of large muscle groups under adequate oxygen supply) and resistance training (strength training using elastic bands). The session structure was as follows: 5 minutes of warm-up, 25 minutes of resistance training, 25 minutes of aerobic exercise, and 5 minutes of cool-down. Sessions were scheduled 2 h after breakfast.

(2) Exercise intensity: According to the American College of Sports Medicine (ACSM) and the American Diabetes Association (ADA), the optimal aerobic exercise intensity for individuals with T2DM is 40–59% of VO₂max or 11–14 on the Borg Rating of Perceived Exertion (RPE) scale, and the resistance training intensity should be 50–69% of one‑repetition maximum (1‑RM). Based on the Chinese Guidelines for the Prevention and Treatment of Type 2 Diabetes and the participants' actual conditions, the intensity of Baduanjin was set to achieve a heart rate of 100–110 beats/min. The MIO heart rate monitor was programmed with upper and lower heart rate limits, which emitted distinct audible signals when exceeded, thereby indicating whether the intensity remained within the prescribed range. For resistance training, the intensity was set at 50–69% of the 1‑RM. As the participants were on antihyperglycemic medications that may affect heart rate, the RPE method was also used to monitor exercise intensity throughout the intervention.

(3) Exercise modality: The intervention group performed moderate-intensity elastic band resistance exercises targeting the major muscle groups of the upper and lower extremities and trunk, together with the complete set of standard Baduanjin movements. One complete set of Baduanjin exercise lasted 12 min, and each session included two complete sets.

(4) Exercise frequency: The World Health Organization recommends aerobic exercise at least three days per week, and for most adults, distributing weekly exercise across three to five days is an effective strategy to meet physical activity recommendations. For resistance exercise, the same muscle groups should be trained with at least one day of rest between the sessions. Accordingly, the participants in the intervention group participated in three supervised sessions per week to ensure an adequate training frequency.

(5) Precautions: ① A liability waiver was signed before each exercise session was conducted. ② Exercise was performed 2 h after meals and never on an empty stomach. Participants wore appropriate and comfortable sportswear and footwear. ④ Trained medical staff were present during all sessions, and sugar cubes and water were readily available to mitigate the risk of hypoglycemia. ⑤ Each participant carried a personal information card that detailed their identity, home address, and emergency contact, which was kept nearby during exercise.

4.3.4 Statistical Analysis

Statistical analyses were performed using R version 4.2.0 (R Foundation for Statistical Computing, Vienna, Austria). Continuous variables are expressed as mean ± standard deviation, and categorical variables as frequencies and percentages. Baseline between-group comparisons were conducted using independent-samples *t*tests for continuous variables and chi-square tests for categorical variables. In the primary analysis, time (baseline vs. post-intervention), group (intervention vs. control), and their interaction were set as fixed effects, with age and sex entered a priori as covariates. To account for within‑subject variability, a random intercept was included for each participant in the model.

**5.** **Key Points and Challenges of the Study**

Based on preliminary research, the following key points and challenges have been identified. The author has accumulated practical experience in preliminary work and has developed corresponding contingency plans.

Key focuses: Demonstrating the dose-response relationship between physical exercise and working memory, with particular emphasis on the implementation and monitoring of the exercise protocol.

Challenges: Effective communication and interaction with participants and ensuring their safety throughout the intervention.

**6.** **Feasibility of the Study**

The author has been engaged in research on physical activity in the elderly population and has established a collaboration with a partner hospital. The hospital is equipped with all the necessary experimental facilities and instrumentation required for this study. Thus, the research foundation, hardware resources, and participant accessibility collectively render this project feasible and promising.

**7.** **Expected Results**

This study is expected to yield positive effects on physiological indicators, psychological status, and self-management behaviors in community-dwelling middle-aged and elderly patients with type 2 diabetes mellitus (T2DM) following a 16‑week intervention combining Baduanjin and elastic band resistance training. The anticipated outcomes are as follows:

(1) Physiological indicators: Compared with the control group, the intervention group is expected to show significant improvements in glycemic parameters, including fasting plasma glucose (FPG), glycated hemoglobin (HbA1c), and homeostatic model assessment of insulin resistance (HOMA‑IR) (P < 0.05). Lipid profiles are also anticipated to improve, with significant reductions in total cholesterol (TC), triglycerides (TG), and low‑density lipoprotein cholesterol (LDL‑C), along with an increase in high‑density lipoprotein cholesterol (HDL‑C). In terms of body composition, body fat percentage and waist‑to‑hip ratio are expected to be notably improved.

(2) Group‑by‑time interaction effects: Repeated‑measures analysis of variance (ANOVA) is predicted to reveal a significant time × group interaction, indicating that the combined exercise intervention is superior to routine health management in improving multiple outcome measures.

In summary, this study is anticipated to confirm that the combination of Baduanjin and elastic band resistance training constitutes an effective, safe, and feasible exercise intervention for middle‑aged and elderly individuals with T2DM in community settings, thereby providing empirical evidence for its wider adoption in community‑based health management.

**8.** **Research Innovations**

(1) Innovation in academic thinking: This project approaches the topic from the perspective of the dose‑response relationship between physical exercise and improvements in body composition, muscle strength, physiological parameters, and glucolipid metabolism in middle‑aged and elderly T2DM patients, emphasizing the integration of theoretical inquiry and empirical investigation.

(2) Innovation in academic perspective: The study concurrently explores dose‑response relationships and underlying physiological mechanisms, rendering the overall argumentation more comprehensive and precise.

(3) Innovation in research methods: In data collection, the study combines instrumental measurements with questionnaire surveys. In research design, it integrates multiple approaches, including bibliometric analysis, observational studies, quasi‑experimental and true experimental designs, with careful attention to logical rigor and completeness, and strives for mutual corroboration among methods.

(4) Innovation in intervention delivery: The intervention is delivered through a collaborative model between a sports university and community hospitals, which facilitates the integration of sports and medicine (i.e., the “sports‑medicine integration” approach) and promotes its practical implementation.

**References**

[1] Guo, J. (2023). Spatiotemporal evolution and influencing factors of population aging in Chinese urban agglomerations: An analysis based on national census data. Journal of Yunnan Minzu University (Philosophy and Social Sciences Edition), 40(4), 84–94.

[2]	WANG L, GAO P, ZHANG M, et al. Prevalence and Ethnic Pattern of Diabetes and Prediabetes in China in 2013 [J]. Jama, 2017, 317(24): 2515-23.

[3]	Q&A: Key points for IDF Diabetes Atlas 2017 [J]. Diabetes Res Clin Pract, 2018, 135: 235-6.

[4]	FEDERATION I D. Worldwide toll of diabetes [Z]. 2021-3-6

[5]	LAM D W, LEROITH D. The worldwide diabetes epidemic [J]. Curr Opin Endocrinol Diabetes Obes, 2012, 19(2): 93-6.

[6]	ZIMMET P Z, MAGLIANO D J, HERMAN W H, SHAW J E. Diabetes: a 21st century challenge [J]. Lancet Diabetes Endocrinol, 2014, 2(1): 56-64.

[7]	SIDDIQUI N I, NESSA A, HOSSAIN M A. Regular physical exercise: way to healthy life [J]. Mymensingh Med J, 2010, 19(1): 154-8.

[8]	SAMPATH KUMAR A, MAIYA A G, SHASTRY B A, et al. Exercise and insulin resistance in type 2 diabetes mellitus: A systematic review and meta-analysis [J]. Ann Phys Rehabil Med, 2019, 62(2): 98-103.

[9]	TEICH T, ZAHARIEVA D P, RIDDELL M C. Advances in Exercise, Physical Activity, and Diabetes Mellitus [J]. Diabetes Technol Ther, 2019, 21(S1): S112-s22.

[10]	KIRWAN J P, SACKS J, NIEUWOUDT S. The essential role of exercise in the management of type 2 diabetes [J]. Cleve Clin J Med, 2017, 84(7 Suppl 1): S15-s21.

[11]	KANALEY J A, COLBERG S R, CORCORAN M H, et al. Exercise/Physical Activity in Individuals with Type 2 Diabetes: A Consensus Statement from the American College of Sports Medicine [J]. Med Sci Sports Exerc, 2022, 54(2): 353-68.

**II. Statistical analysis plan**

All statistical analyses will be performed using R version 4.2.0 (R Foundation for Statistical Computing, Vienna, Austria), with two‑sided P < 0.05 considered statistically significant, and will follow the intention‑to‑treat principle, including all randomized participants in their originally assigned groups. Continuous variables will be summarised as mean (standard deviation) and categorical variables as counts (percentages); baseline between‑group comparisons will be conducted using independent‑samples *t*‑tests or Mann‑Whitney U tests for continuous variables and chi‑square or Fisher’s exact tests for categorical variables, as appropriate. To evaluate the intervention effect over time, linear mixed‑effects models will be fitted with time (baseline versus 4‑month follow‑up), group (intervention versus control), and their interaction as fixed effects, with age and sex entered a priori as covariates, and a random intercept for each participant to account for within‑subject repeated measurements; the primary inference will be based on the group‑by‑time interaction coefficient, reported with its 95% confidence interval and P‑value. Missing data, which are anticipated to be minimal, will be handled under the missing‑at‑random assumption using multiple imputation by chained equations with predictive mean matching, generating 10 imputed datasets and 50 iterations each, and the results from the mixed models applied to each imputed dataset will be pooled according to Rubin’s rules. All secondary outcomes will be analysed with the same mixed‑model approach without correction for multiple comparisons, given their exploratory nature, and results will be interpreted cautiously.

**III. Prespecified primary and secondary outcome measures**

**Primary Outcome Measures**

**Glycemic Control**

**Fasting Plasma Glucose (FPG, mmol/L):** Measured using an automated clinical chemistry analyzer after at least 8 hours of fasting.

**Glycated Hemoglobin (HbA1c, %):** Measured using a high-performance liquid chromatography (HPLC) system.

**Body Composition：**Weight (kg); Body Mass Index (BMI, kg/m²); Waist Circumference (WC, cm); Hip Circumference (HC, cm). Measured using a bioelectrical impedance analyzer (InBody 770).

**Muscle Strength:** Handgrip Strength (HGS, kg). Measured bilaterally using a digital handgrip dynamometer (T.K.K.5401). Both left and right handgrip strength will be assessed.

**Secondary Outcome Measures**

**Blood Pressure:** Systolic Blood Pressure (SBP, mmHg)**;** Diastolic Blood Pressure (DBP, mmHg).

Measured using an automated electronic blood pressure monitor (Omron HEM-907) with standardized procedures.

**Lipid Profile:** Total Cholesterol (TC, mmol/L); Triglycerides (TG, mmol/L); High-Density Lipoprotein Cholesterol (HDL-C, mmol/L); Low-Density Lipoprotein Cholesterol (LDL-C, mmol/L); Very Low-Density Lipoprotein Cholesterol (VLDL-C, mmol/L)

Measured using an automated clinical chemistry analyzer.
