# Supplementary material for: Effectiveness of community—based Baduanjin combined with resistance training on cardiometabolic risk factors in older adults with type 2 diabetes: a 16-week randomized controlled trial
Source: Front Endocrinol (Lausanne). 2026 Aug 27;17:1930328. doi: 10.3389/fendo.2026.1930328 (PMC13557981; doi:10.3389/fendo.2026.1930328)
Supplement: Supplementary file 2 [file SupplementaryFile1.doc]

**Combined Baduanjin and Elastic-band Training Improves Cardiometabolic Risk Profile in Older Adults with Type 2 Diabetes: A 16-Week Randomized Controlled Trial**

**Supplementary Material**

I. CONSORT 2025 checklist of information to include when reporting a randomised trial
II. Study protocol
III. Intervention protocol
IV. Primary and Secondary outcomes analysis

Supplementary Material II-Study protocol

Figure 1. Schematic representation of participants in the screening, randomization, and interventions

Enrolment

Eligibility assessment

Not eligible

Randomization(n≥90）

Allocation

Allocate to Regular Health Management

Allocate to self-management group

Intervention

Complete post-intervention assessment

Complete post-intervention assessment

Post-intervention follow-up

Complete post-follow-up assessment

Complete post-follow-up assessment

Date analysis

Date analysis

Supplementary Material III-Intervention protocol

Part A. Session Contents of Regular Health Management

| Module | Topic | Topic content |
| --- | --- | --- |
| Core  Education | Lifestyle goal  setting | a) Overview of T2DM and lifestyle-related risk factors b) set individualized management targets |
| (30min) | Dietary  management | a) regular meal timing and planned snacks b) reduce sugar-sweetened beverages and refined carbohydrates |
|  | Physical activity | a) FITT principles (frequency, intensity, time, type) b) aerobic plus resistance training c) safety considerations |
|  | Smoking and  alcohol | a) Health risks of smoking and cessation strategies b) principles of alcohol moderation |
|  | Sleep and daily  routine | a) Establish regular sleep–wake schedules b) reduce late nights and nighttime snacking c) stress reduction and brief relaxation techniques |
|  | Psychosocial  support | a) Identify and address emotional distress b) enhance self-efficacy c) mobilize family/peer support |
| Thematic  Education  (30min) | Appropriate  medication use | a) Principles of glucose-lowering therapy b) timing and coordination with meals and physical activity |
|  | Self-monitoring  of blood glucose | a) recommended timing and frequency b) correct meter use and sources of measurement error c) logging and basic interpretation |
|  | Hypoglycemia  prevention  and management | a) Identify high-risk situations b) recognize typical symptoms c) nocturnal hypoglycemia precautions |
|  | Daily care | a) Daily foot checks and appropriate footwear b) skin and oral care c) wound care precautions, infection warning signs, and when to seek care. |
|  | Cardiovascular  risk factor  management | a) Management of blood pressure, lipids, weight, and waist   circumference   b) dietary salt/fat reduction and physical activity c) reinforce smoking cessation |
|  | Prevention and  screening of chronic complications | a) Prevention of retinopathy, nephropathy, and neuropathy b) annual screening items and recommended intervals c) recognition of red-flag symptoms |

Note: The standard management consists of four sessions (one per month, lasting for 4 months); each session includes core education (lifestyle) and thematic education (disease management).

Part B. Session Contents of self-management group

| Session | Content |
| --- | --- |
| Warm-up(6min) | a) Light aerobic exercise (e.g., marching in place or arm circles) b) Dynamic stretching (e.g., leg swings, arm swings) c) Focus on increasing heart rate and joint mobility |
| Baduanjin(24min) | 1. Hold the sky with both hands to adjust the triple focus. 2. Bow left and right like shooting eagles.   3. To regulate the spleen and stomach must be single lifted.  4. Look back to treat damaged organs.  5. Shake your head and tail to get rid of psychological pressure.  6. Climbing feet with both hands to strengthen the kidney and waist.  7. Grow fists to increase strength.  8. Behind the 7 tiptoes to eliminate all diseases. |
| Resistant band  training (24min) | a) Seated Chest Press: With the band behind the back, extend arms forward while keeping the core engaged. Targets pectorals and triceps. b) Seated Shoulder Press: Place the band under the thighs, press arms upward. Targets deltoids and biceps. c) Seated Reverse Fly: Hold the band at chest height and pull apart, engaging posterior deltoids and rhomboids. d) Seated Arm Curl: Curl the forearms toward the upper arms, targeting biceps. e) Seated Knee Raise: Raise knees towards the chest, activating the quadriceps. f) Seated Hip Abduction: Push feet apart against the band to engage hip abductors and improve pelvic stability. g) Seated Knee Extension: Extend one leg forward, engaging the quadriceps. h) Seated Leg Press: Press foot forward, targeting quadriceps, hamstrings, and glutes. i) Seated Knee Flexion: Bend the knee towards the chest, strengthening the hamstrings. j) Standing Heel Raise: Rise onto toes, engaging the calves. |
| Cool-down(6min) | 1. Hamstring Stretch: Stretch the back of the leg by reaching for the toes of an extended leg. 2. Quad Stretch: Pull the foot towards the glutes, stretching the quadriceps. 3. Shoulder Stretch: Pull one arm across the body to stretch the shoulder. 4. Calf Stretch: Press the heel of one foot towards the floor, stretching the calf. |

Note: Each exercise is performed slowly with controlled breathing; Participants will perform each exercise in a seated position with the resistance band securely anchored to either the back or legs.

**Supplementary Material IV-Primary and secondary outcomes analysis**

|  | **Control(n=63)** | | **Intervention(n=60)** | | **Group effect** | | **Time effect** | |
| --- | --- | --- | --- | --- | --- | --- | --- | --- |
|  | **P-value** | **Cohen’s d** | **P-value** | **Cohen’s d** | **Estimate(95%CI)** | **P-value** | **Estimate(95%CI)** | **P-value** |
| Primary outcomes | | | | | | | | |
| weight | 0.026 | -0.205 | <0.001 | -0.579 | 1.59(-2.40, 5.57) | 0.437 | -1.99(-2.60, -1.38) | <0.001 |
| BMI | 0.039 | -0.190 | <0.001 | -0.569 | 0.45(-1.05, 1.95) | 0.555 | -0.73,-0.96, -0.50) | <0.001 |
| WC | 0.001 | -0.302 | <0.001 | -0.638 | -1.24(-4.08, 2.32) | 0.498 | -2.24(-2.87, -1.62) | <0.001 |
| HC | 0.273 | -0.100 | <0.001 | -0.310 | -1.33(-3.78, 1.13) | 0.291 | -1.13(-1.79, -0.48) | <0.001 |
| FPG | 0.151 | 0.132 | 0.026 | -0.205 | -0.53(-1.28, 0.23) | 0.173 | -0.50(-0.94, -0.07) | 0.026 |
| HbA1c | 0.119 | 0.143 | 0.007 | -0.247 | -0.11(-0.55, 0.33) | 0.631 | -0.25(-0.43, -0.07) | 0.007 |
| LHGS | <0.001 | 0.356 | <0.001 | 0.839 | 0.20(-2.21, 2.61) | 0.872 | 3.44(2.71, 4.18) | <0.001 |
| RHGS | 0.002 | 0.289 | <0.001 | 1.012 | -0.86(-3.58, 1.85) | 0.535 | 5.20(4.29, 6.12) | <0.001 |
| Secondary outcomes | | | | | | | | |
| SBP | <0.001 | -0.407 | <0.001 | -0.616 | 0.82(-3.83, 5.47) | 0.731 | -8.47(-10.92,-6.02) | <0.001 |
| DBP | <0.001 | -0.407 | <0.001 | -0.620 | -0.33(-3.85, 3.20) | 0.857 | -6.42(-8.26, -4.57) | <0.001 |
| TC | 0.819 | 0.021 | 0.447 | -0.069 | 0.40(-0.10, 0.90) | 0.119 | -0.12(-0.42, 0.18) | 0.447 |
| TG | 0.780 | -0.025 | 0.037 | -0.192 | -0.30(-0.80, 0.20) | 0.246 | -0.32(-0.62, -0.02) | 0.037 |
| HDL-C | 0.987 | 0.001 | 0.159 | 0.129 | 0.09(-0.01,0.19) | 0.053 | 0.04(-0.02, 0.10) | 0.159 |
| LDL-C | 0.717 | 0.033 | 0.813 | -0.021 | 0.22(-0.18, 0.62) | 0.277 | -0.03(-0.29, 0.22) | 0.813 |
| VLDL-C | 0.334 | -0.088 | 0.035 | -0.193 | 0.11(-0.02,0.23) | 0.091 | -0.10(-0.19,-0.01) | 0.035 |

Note: BMI Body mass index, WC waist circumference, HC hip circumference, FPG Fasting blood glucose, HbA1c Glycosylated hemoglobin, LHGS Left handgrip strength, RHGS Right handgrip strength, SBP Systolic blood pressure, DBP Diastolic blood pressure, TC Total cholesterol, TG	Triglyceride, HDL-C High-density lipoprotein cholesterol, LDL-C Low-density lipoprotein cholesterol, VLDL-C Very low-density lipoprotein cholesterol.
